# Vision-Sensor-Assisted Probabilistic Localization Method for Indoor Environment

**DOI:** 10.3390/s22197114

**Published:** 2022-09-20

**Authors:** Hui Shi, Jianyu Yang, Jiashun Shi, Lida Zhu, Guofa Wang

**Affiliations:** 1 School of Mechanical Engineering and Automation, Northeastern University, Shenyang 110819, China; 2 China Coal Technology and Engineering Group, Beijing 100013, China

**Keywords:** AMCL, indoor localization, robot kidnapping problem, premature convergence

## Abstract

Among the numerous indoor localization methods, Light-Detection-and-Ranging (LiDAR)-based probabilistic algorithms have been extensively applied to indoor localization due to their real-time performance and high accuracy. Nevertheless, these methods are challenged in symmetrical environments when tackling global localization and the robot kidnapping problem. In this paper, a novel hybrid method that combines visual and probabilistic localization results is proposed. Augmented Monte Carlo Localization (AMCL) is improved for position tracking continually. LiDAR-based measurements’ uncertainty is evaluated to incorporate discrete visual-based results; therefore, a better diversity of the particle can be maintained. The robot kidnapping problem can be detected and solved by preventing premature convergence of the particle filter. Extensive experiments were implemented to validate the robustness and accuracy performance. Meanwhile, the localization error was reduced from 30 mm to 9 mm during a 600 m tour.

## 1. Introduction

Autonomous navigation requires the mobile robot to localize itself autonomously in a dynamic environment. To obtain the current location on the known map, various techniques and algorithms have been developed. Normally, the localization problem is solved at three levels, position tracking, global localization, and the robot kidnapping problem [1]. As far as practical application is concerned, besides solving the three sub-problems, real-time performance and accuracy also need to be considered. Approaches applying various sensor modalities are challenged by specific problems. On the one hand, Wireless-Sensor-Network (WSN)-based methods utilizing Ultra Wide Band (UWB) [2], WiFi [3,4], and Bluetooth Low Energy (BLE) [5]) can localize the robot with the Received Signal Strength Indicator (RSSI), which is unique at specific location. Such methods rely on Access Point (AP) deployment, and the accuracy is sensitive to the surrounding noise [6,7]. On the other hand, exteroceptive-sensor-based methods determine the robot’s states (including position and orientation) by perceiving the surroundings with sensors mounted on the robot. Perceptual data are fused with algorithms such as the Kalman Filter (KF) and its extensions (extended Kalman filter and unscented Kalman filter [8]), grid localization, and the Particle Filter (PF). Among them, the PF is a widely used method with a multi-modal probabilistic density function (pdf); it is a prevalent approach to the nonlinear and non-Gaussian state estimation.

**Figure 1:**
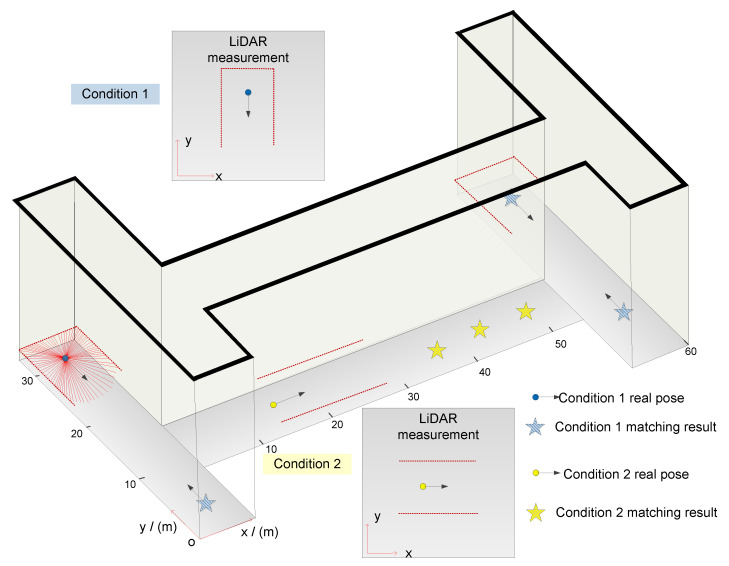
Confusing measurement of range finder perception in repetitive environment.

AMCL is a typical PF developed from Monte Carlo Localization (MCL) to incorporate various sensor modalities. MCL is a recursive form of the Bayesian Filter (BF), since the probability of the true pose is described with a fixed number of particles, the real-time performance in a large area is challenged, and it cannot deal with the robot kidnapping problem. Hence, AMCL is proposed by fusing an adaptive technique with MCL, and the particle number varies when solving different sub-problems. For instance, position tracking is resolved with minimum particles, and when it comes to global localization or the robot kidnapping problem, more particles are applied to cover potential states [9]. However, conventional AMCL is faced with the robot kidnapping problem during long-term performance in symmetrical environments with repetitive features. Taking the environment shown in Figure 1 as an example, the algorithm models the motion model with odometers and perceives the surroundings with LiDAR. When the accumulated errors of the odometers are too high, the re-localization mechanism can be aroused by a sudden turn or dynamic environment. Since the adaptive mechanism of conventional AMCL determines the particle number only considering the LiDAR measurement likelihood [10,11,12], this directly brings up the particle premature convergence problem in symmetrical environments.

By investigating the underlying relationship between LiDAR measurement and the algorithm, this paper reveals the causes of catastrophic problems in global localization and the robot kidnapping problem in a specific environment. General measurements are shown in the two conditions, since scans are applied to match with the existing map, and the limited distance measurements of LiDAR bring up several matching results. For instance, the circles represent the robot’s real pose, and the perceived surroundings are demonstrated in Condition 1 and Condition 2, respectively. Then, to correct the probabilistic estimation, the observation is used to match with the known map. In both conditions, there are a few confusing matching results due to the repetitive features in the environment. The robot cannot determine which location is correct until a distinguishable feature is observed. During the autonomous navigation mission in Condition 1, the motion is chaotic before arriving at the T-junction, and there is no guarantee that the perception is correct, so matching the LiDAR observation to the map is not reliable [13]. When the robot is in a long corridor, as demonstrated in Condition 2, a similar local submap can be obtained at many locations, as shown by the yellow stars. Therefore, once the right particle is determined during this stage, the algorithm is challenged by the problem of particle degeneracy. To obtain a better diversity of the particles, resampling methods were developed in previous works. Lin et al. proposed to improve FastSLAM with an adaptive bat-inspired resampling [14]. Manizheh et al. preserved the particle diversity by optimizing the proposal distribution [15]. More resampling techniques were introduced in [9,16,17]. In previous works, approaches have been proposed to solve the robot kidnapping problem, but the accuracy and long-term performance were not considered [18,19].

To address the above challenges, this paper presents a low-cost and convenient method to deal with the global localization problem and robot kidnapping problem in a specific environment. The conventional LiDAR-based AMCL [10] is improved to incorporate the discrete visual observation efficiently. The contributions of this work are as follows:

(a) The resampling stage of the conventional AMCL is improved to reposition particles in regions determined by visual sensors. The discrete visual localization results are tightly coupled with the filter. Compared to LiDAR-based AMCL, the proposed method is more robust to repetitive environments, while the efficiency property is not affected.
(b) The correction mechanism can be aroused automatically without preventing the proper continuous operation of the robots. Besides obtaining high-level accuracy, the robot kidnapping problem can also be avoided and solved. Since it is parallel to AMCL, even the visual part fails temporarily, and the position tracking can be maintained when landmarks are not available.
(c) Abundant experiments were implemented to validate that the framework is endowed with high accuracy and efficiency during long-term performance. The improved AMCL can be generalized to other systems with different forms of data as well.

The remainder of this paper is organized as follows. Section 2 introduces related works. Section 3 introduces the improved AMCL assisted by the absolute method. Experiments are presented in Section 4. Finally, Section 5 concludes this work.

## 2. Related Works

The indoor localization problem has been explored much in previous works, and algorithms were developed from different views separately, including accuracy [20,21], global localization [22], and the robot kidnapping problem [23]. WSN-based methods have been mainly improved for accuracy. Sandra et al. resolved the localization problem in multi-room indoor environments with UWB, trilateration, and fingerprinting combined to cope with the Received Signal Strength Indicator (RSSI). The specific AP deployment strategy limits the method suitable for small environments [2]. Yu et al. combined BLE with other sensors for 3D localization [24]. Such RSSI-based methods are vulnerable to fluctuating signals and AP deployment. Aiming to improve the accuracy, Ullah et al. provided a reference research on the effect of various factors [25]. Bharadwaj and Koul proposed to mitigate the unreliable nature of the RSSI, which is easily plagued by surrounding noise [26]. It can be seen that the above methods are limited by AP deployment and signal stability; this nature makes them unsuitable for the specific environment shown in Figure 1.

To obtain a convenient localization framework, exteroceptive sensors such as LiDARs and cameras have been widely used. To be exact, the performance has been evaluated by balancing the accuracy, the capacity to solve global localization, and the robot kidnapping problem. Visual–LiDAR-fusion-based methods have been extensively applied for SLAM [27]. Li et al. proposed a high-precision positioning method to improve the localization accuracy by fusing the inertial measurement unit (IMU), monocular camera, and LiDAR. The system consisted of IMU–3D laser data filtering and IMU–monocular data filtering. The federated filtering for the multi-sensor integrated navigation system was based on the KF. The approach was implemented to track the robot’s position [22]. In [20,21], the accuracy of LiDAR odometry and visual odometry was studied. Though they could localize the robot continuously, the accumulated error and computation burden made it unsuitable for long-term operation.

Wang et al. utilized a 2D LiDAR to solve the global localization problem in a probabilistic way [28]. The proposed algorithm utilizes a qualitative motion model, and the capability to cope with global localization was verified in the experiments, while the convergence speed and the robustness to dynamic objects were left to be improved. Guan et al. incorporated the Kullback–Leibler Divergence (KLD) sampling with the Random Finite Set (RFS) model to MCL. Doppler–azimuth radar was applied to provide global pose, and their method was tested in an area of 12 m × 12 m with seven landmarks [29]. Chen et al. proposed heuristic Monte Carlo for local and global localization. The prior map obtained by radar was processed by an image-processing algorithm, then the Discrete Hough Transform (DHT) was implemented to match the visual features. During the localization stage, the particles obtained by the matching results were used as the heuristic distribution of MCL [30].

The kidnapping problem, which is considered as the most difficult one, is usually resolved in two steps, kidnap detection and recovery. Chien et al. worked with the global localization problem in symmetrical environments, and the premature convergence of MCL for such occasions was investigated. Multi-Objective Particle Swarm Optimization (MOPSO) was introduced to detect the failure and to resample the particles with balanced weights, and the Pareto front was incorporated with MCL. The algorithm was proven with simulations, while the run-time performance, which is related to practical applications, was left to be improved [19]. Campbell and Whitty aimed to detect all kidnapping events during the autonomous navigation of a robot. Various metrics were evaluated in the combined detector, then the most suitable metrics were determined by optimal thresholds. The detection accuracy relied on the classification of the kidnapping problem and the chosen thresholds [31]. Su et al. solved the kidnapping problem of AMCL by integrating a LiDAR and camera. To retrieve pose proposals directly, the scene was represented by a grid map and Gist descriptors. Image retrieval and keyframe clustering were applied to refine the robot’s pose in the LiDAR likelihood framework. The continuous localization was completed by the probabilistic method, and the visual localization was applied to supply global poses for position tracking [23]. Yilmaz and Temeltas improved Self-Adaptive Monte Carlo (SAMCL) by making the algorithm suitable for AGVs equipped with 2D or 3D LiDARs. The experiments showed that when unexpected collisions happened, the recovery needed 14-16 s for global localization [12]. Hence, a balanced approach capable of solving the practical autonomous localization in the specific environment shown in Figure 1 is still needed.

## 3. Proposed Method

**Figure 2:**
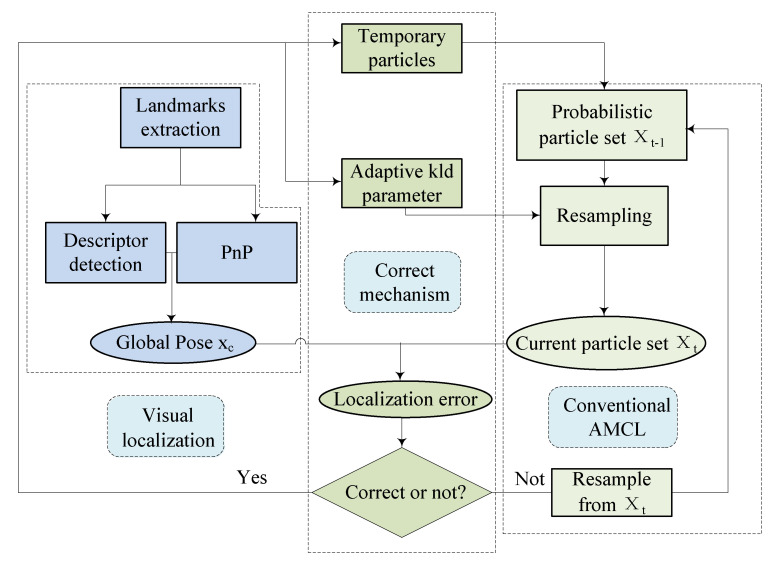
Architecture of the proposed method.

The proposed method locates the robot globally by incorporating visual observation with the filter calculation. The architecture mainly includes three parts, as shown in Figure 2. Firstly, the visual localization calculates the discrete global pose with the aid of landmarks. To improve the efficiency and robustness of the image processing, Histogram of Oriented Gradients (HOG) descriptors [32] and k-Nearest Neighbor (KNN) [33] are applied for real-time descriptor detection. Once a target is detected, the corresponding global location can be obtained. Perspective-n-Points (PnP) is implemented to calculate the pose $c_{t}$ relative to the markers with the dimensions known. After that, the KLD sampling part is exploited to incorporate the visual localization results in a probabilistic manner. Specific particles are generated based on the visual localization and added to the existing particle set maintained by the conventional AMCL, and the threshold of the particle number can be more reasonable to prevent the premature convergence problem in symmetrical environments. Therefore, the uncertainty can be evaluated considering both visual and LiDAR measurements. The correction mechanism is aroused once the Euclidean distancebetween the conventional AMCL estimation and visual localization result is higher than the threshold.

### 3.1. Improved AMCL Framework

The parameters utilized to introduce the algorithm are listed in Nomenclature. The improved AMCL can be divided into 4 steps: initialization, updating sensor observation, resampling, and estimation considering visual perception. The framework is shown in Algorithm 1.
**Algorithm 1** Improved probabilistic algorithm**Input:** 
previous state and sensor observations (Initialization)**Output:** 
threshold of particle number [10];1:$x_{t}^{\left[i\right]}$ = sample motion model($u_{t},x_{t}^{\left[i\right]}$);2:$\omega_{t}^{\left[i\right]}$ = likelihood field($z_{t},x_{t}^{\left[i\right]},m$), (Update);3:$\chi_{temp}$ = $\chi_{temp}$ +<$x_{t}^{\left[i\right]},\omega_{t}^{\left[i\right]}$>, (Resampling);4:**if** d>σ  **then**5:    generate particle set $C_{t}$ center on $x_{c}$, (Incorporate visual observation)6:    $\chi_{c}$= <$C_{t}$,$\omega_{t}^{\left[i\right]}$>7:**else**$\chi_{c}$= random particles8:**end if**9:add $\chi_{c}$ to $\chi_{t}$ with probability ($0,1-\frac{\omega_{fast}}{\omega_{slow}}$)

Initialization: As a recursive method, the algorithm is initialized with assumptions. The conventional AMCL distributes particles on the whole map with an average weight for global localization, then the current pose needs to be input or calculated for position tracking. For such occasions, the robot needs to move a little to obtain the surroundings’ information. However, in the symmetrical environment shown in Figure 1, the initialization is time-consuming and confusing. Aided by camera observation, the visual localization results can be applied to determine the initial pose for position tracking.

Update and resampling: In detail, $u_{t}$ and $z_{t}$ are recursively utilized to update the state for tracking. Assume there are *M* particles in the initial set. The robot’s pose is calculated in a probabilistic manner. $bel(x_{t})$ of the set $\chi_{t}$ is calculated by Equation (1). $x_{t}^{\left[i\right]}$ denotes the pose of the *i*th particle at the *t*th moment, as shown in Equation (2), and it is calculated based on the previous state and $u_{t}$.

(1) $$bel\left(x_{t}\right)=p\left(x_{t}|z_{1:t},u_{1:t}\right)$$

(2) $$x_{t}^{\left[i\right]}=p(x_{t}|x_{t-1}^{\left[i\right]},u_{t}),i=1,\ldots ,M.$$

Then local measurements of the LiDAR are applied to match with the known map, which is represented as importance factor $\omega_{t}^{\left[i\right]}$ calculated by Equation (3). In this stage, the samples with low $\omega_{t}$ are eliminated. In the environment with repetitive features or even one that is symmetrical in structure, the wrong samples share similar measurements to the correct one. When the algorithm becomes trapped in the wrong local optimum, premature convergence is induced.

(3) $$\omega_{t}^{\left[i\right]}=p(z_{t}|x_{t}^{\left[i\right]})$$

Incorporating visual observation: To maintain better diversity, visual localization results are considered as the reference. When the Euclidean distance *d* between visual localization result $x_{c}$ and the current estimation of AMCL $x_{t}$ exceeds the threshold σ, specific particles are generated to improve the reliability of the measurement before evaluating the uncertainty. Theoretically, by distributing the particles in a wider area, one can obtain better diversity. To increase the weight of visual-based localization, the number of specific particles is generally more than the existing set. Then, assigning $\omega_{avg}$, calculated by Equation (4), specific particles are preserved in the set $\chi_{c}$. For instance, when the area is defined as a square whose radius is *r*, specific particles are generated centered on $x_{c}$ with a Gaussian distribution.

(4) $$\omega_{avg}\approx p\left(z_{t}|z_{1:t-1},u_{1:t},m\right)$$

### 3.2. Visual-Based Global Localization

**Figure 3:**
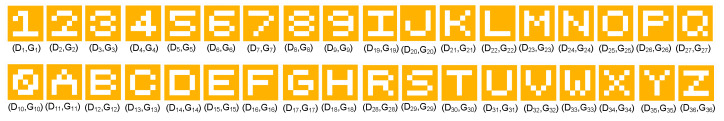
Markers deployed in the environment.

Aside from unexpected collisions and manual intervention, the symmetrical nature of the environment is also a non-negligible factor that results in high measurement uncertainty. As plausible particles share similar weights in a long corridor with few features, once the right pose is eliminated, the algorithm may keep track of the wrong pose. Vision sensors with redundant information can solve such ambiguities. The RoboMaster S1 vision markers with known dimensions were deployed in the environment, as shown in Figure 3. Once a marker is perceived, the robot’s absolute pose relative to the marker can be calculated. Three stages are included: feature extraction, descriptor recognition, and the PnP algorithm.

The observed images are described with the RGB color palette, consisting of Red (R), Green (G), and Blue (B). Then, the grayscale and gamma transform are imposed to extract the HOG descriptors efficiently. All the RGB images are described with the Hue, Value, Saturation (HSV) parameters. Assuming that $g_{x}$ and $g_{y}$ are gradients for horizontal and vertical edges, the amplitude M(x,y) and angle θ can be expressed by the formulas in Equations (5) and (6).

(5) $$M\left(x,y\right)=\sqrt{{g_{x}}^{2}+{g_{y}}^{2}}$$

(6) $$\theta ={tan}^{-1}\left(\frac{g_{y}}{g_{x}}\right)$$

**Figure 4:**
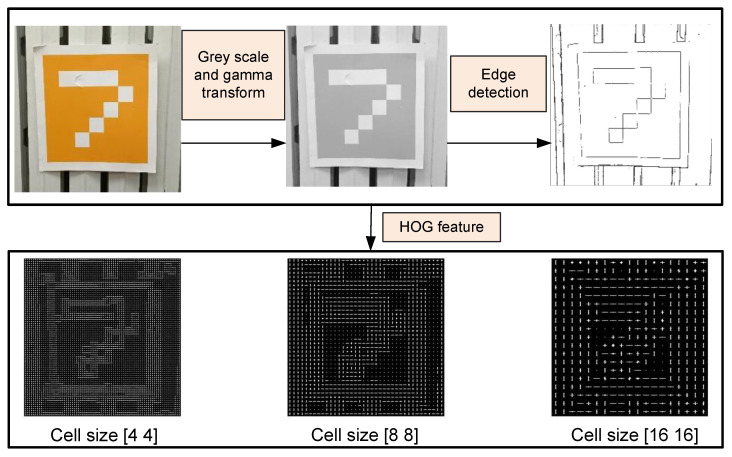
HOG features with different cell sizes.

Then, a window *I*, which consists of cells, is applied to find the gradient, and each cell has a local histogram of the 1D gradient directions. To compute the cell gradient histogram, *M* and θ are considered as the horizontal and vertical axis of the histogram, respectively, and each pixel in the cell is mapped. The detected HOG features with different cell sizes are presented in Figure 4.

The extracted descriptors are recognized by KNN. Denote the training dataset as $T=\left(q_{i},D_{i}\right)$. Each marker is labeled with a global position $G_{i}$ on the grid map and a signature $D_{i}$. The finite set of signatures is denoted as $\varrho =D_{1},D_{2},\cdots ,D_{N}$; let Ξ denote the domain of instance $q_{i}$. The markers applied are presented in Figure 3. The images of the markers observed from different views are preserved in the training dataset. KNN obtains the set $N_{K}\left(x\right)$ by calculating the similarity between the query $d_{i}$ and training samples $D_{i}$ with Equation (7). In the experiment, each marker was sampled 500 times, and the accuracy peaked at 93.25% with k=20.

(7) $$L_{p}\left(d_{i},D_{j}\right)={\left(\sum_{k=1}^{n}{\left|{d_{i}}^{k}-{D_{j}}^{k}\right|}^{p}\right)}^{\frac{1}{p}}$$

**Figure 5:**
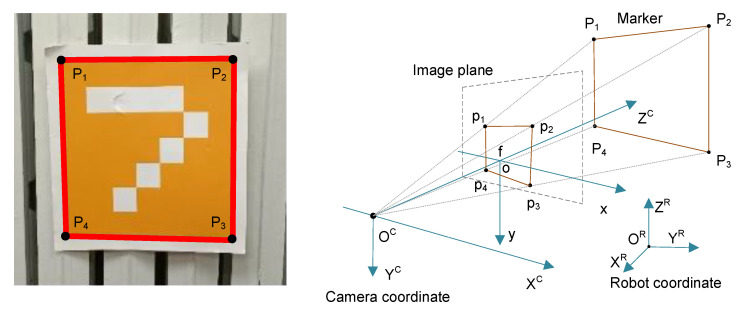
Global localization with markers.

With a recognized acquisition, $G_{i}$ can be determined, as shown in Figure 5. To calculate the camera’s relative pose in the markers’ coordinate system, four 3D corners of the marker’s outer frame were extracted, represented by $P_{i}\left(i=1,2,3,4\right)$, and the specific point of this expression can be expanded as $P_{i}={\left[X_{i},Y_{i},Z_{i}\right]}^{T}$. To calculate the depth information of the monocular camera, markers and the camera are at the same height. Then, the distance *d* between the camera and marker can be obtained by Equation (8). Finally, the problem is formulated and solved by the P4P problem [34].

(8) $$d=\frac{P_{1}P_{2}f}{p_{1}p_{2}}$$

### 3.3. Correction Mechanism with Visual Localization Results

LiDAR-based AMCL corrects the pose according to the matching result of the likelihood field. The proposed approach corrects the pose with both LiDAR and visual measurements. From the view of premature convergence, the solution is analyzed from uncertainty assessment and the calculation of the particle number.

The uncertainty level is assessed from two aspects. The general method is to monitor the measurement probability $p(z_{t}|z_{1:t-1},u_{1:t},m)$. Since $\omega_{t}^{\left[i\right]}$ is also formulated based on $z_{t}$, the average weight of $\chi_{t}$ can be considered as the stochastic estimation of the measurement probability, as described in Equation (4). Meanwhile, $\omega_{fast}$ and $\omega_{slow}$ are maintained to relieve the impact of accidental sensor noises. Here, $\alpha_{fast},\alpha_{fast}$ satisfies $0\leq \alpha_{slow}\ll \alpha_{fast}$. When the robot runs smoothly, the two factors have the following relationships: $\omega_{fast}\geq \omega_{slow}$ or $\omega_{fast}\approx \omega_{slow}$. Occasions such as the robot being manually moved to another room can result in a sudden change of $z_{t}$, then $\omega_{fast}\leq \omega_{slow}$. This means the perceived surroundings cannot be matched to the local map estimated by the algorithm. To cope with the high uncertainty, more particles are distributed to cover possible areas. For example, in the face of the initialization and global localization stages, more particles are needed to cover the whole map, and the uncertainty is high, while few particles are applied for position tracking with a lower uncertainty. The algorithm is unable to correct the estimation result directly. In high uncertainty occasions, the particle number threshold is improved and more random particles are supplied for matching. However, there is no guarantee that it can relocate the particles successfully in symmetrical environments. In comparison, the deviation can be detected and corrected directly with the help of visual observations. With specific particles added, the visual-based locations are incorporated with grid localization by adjusting the belief interval.

The particle number is calculated by the KLD [10]. Since a high uncertainty level means a dispersive distribution, correspondingly, more state spaces are occupied by particles. Assume κ represents the number of nonempty state spaces; $M_{\chi }$ can be calculated by Equation (9). With ϵ and δ, the value of $z_{1-\delta }$ can be obtained by the standard normal distribution.

(9) $$M_{\chi }=\frac{\epsilon }{\kappa }{\left(1-\frac{\kappa }{9}+\left(\sqrt{\frac{\kappa }{9}}z_{1-\delta }\right)\right)}^{3}$$

## 4. Experiment Results

**Figure 6:**
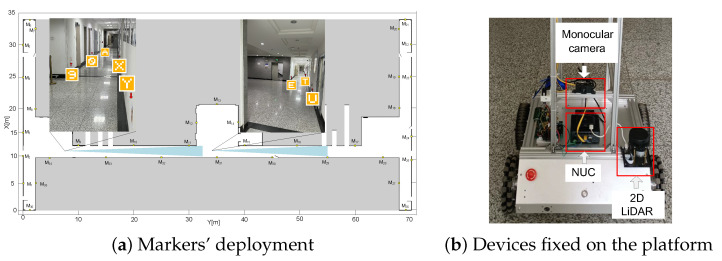
Experiment environment and platform.

The proposed method was tested with a wheeled robot equipped with a monocular camera (S2A17) and a 2D LiDAR (RPLiDAR A1); as shown in Figure 6b, two odometers were mounted on the drive wheels for motion model sampling. The algorithm was run on the onboard computer NUC10I7FNH (Intel6-core I7). The robot ran autonomously on the map shown in Figure 6a; the white space is the corridor in which it is free to move, and the black lines are the walls, while the gray space is unknown. Prior information included a grid map and a dataset consisting of the markers’ signatures and global poses. Vision markers were attached at the same height as the camera. The practical application of the proposed method was examined using the following metrics. Position tracking and global localization performance were analyzed in experiments.

### 4.1. Position Tracking

**Figure 7:**
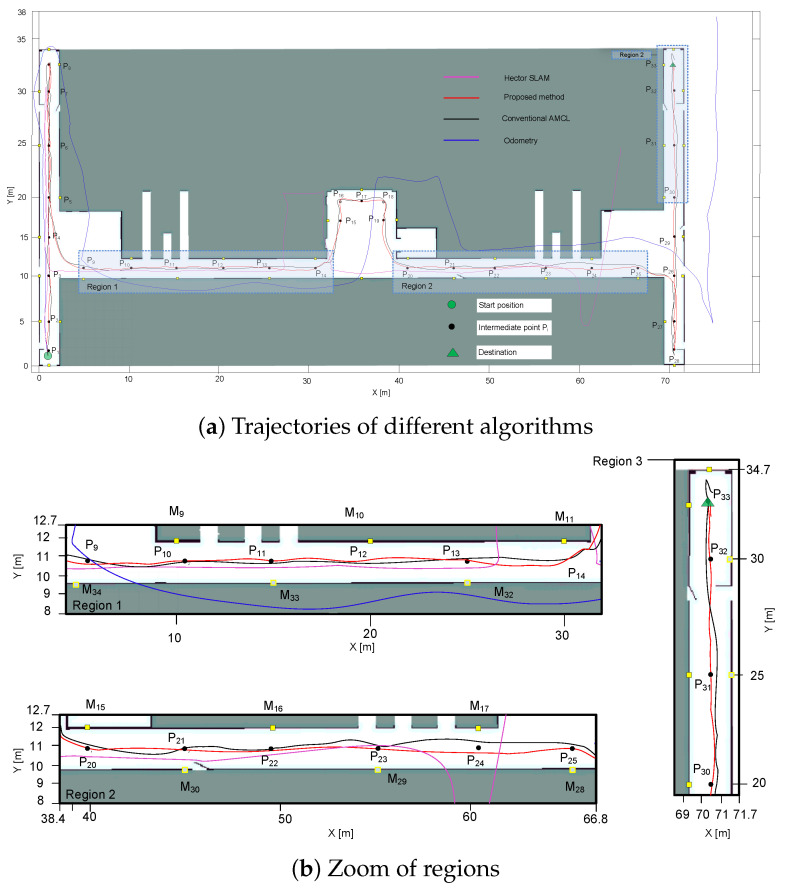
Position tracking comparison of different algorithms.

The position tracking experiment was implemented with an autonomous navigation mission. The autonomous navigation aimed to compare the proposed method with the conventional AMCL. The robot drove one lap on the map, which was formulated by $P_{1}-P_{33}$, sequentially, and $P_{33}-P_{25}-P_{9}-P_{1}$, with an average speed of 30 cm/s. Note that only the markers on the left side of the robot can be perceived. Rather than comparing with a continuous ground truth, the localization accuracy was demonstrated based on the localization results at intermediate points $P_{i}$. In Figure 7a, the results of the proposed method and the conventional AMCL are shown in red and black solid lines; the odometers of the robot were applied to formulate the odometry trajectory. shown in the blue solid line. The pink solid line represents the Hector SLAM results introduced in [35]. The algorithm ran from $P_{1}-P_{33}$ utilizing 2D LiDAR, and it failed to track the robot’s position in the experiment.

**Figure 8:**
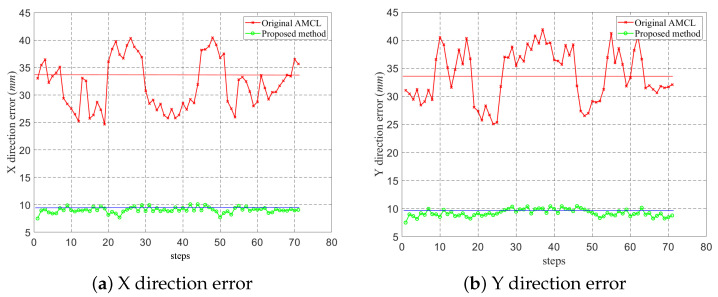
Localization error of different algorithms.

Intermediate points were the targets that the robot should arrive during the mission. Both conventional AMCL and the proposed method can track the robot’s position successfully. However, as shown in Figure 7b, the trajectory of the proposed method was more in accordance with the predefined targets. As presented in Region 3, there was an obvious deviation along the movement direction at point $P_{33}$. To evaluate the accuracy of the algorithms in more detail, both methods were examined five times, and each time, 71 localization results at intermediate points during the mission were recorded. The localization errors in both directions are analyzed in Figure 8.

The average localization errors at the known points are shown in Figure 8. As the environment is narrow, the robot can only run in the horizontal or vertical direction and the angular precision is hardly affected, so the orientation error was not included. From Figure 8, the error of the conventional AMCL is higher than the proposed method. Alternatively, in Table 1, the RMSE values of the two methods are compared. The results showed that the performance of the proposed algorithm was more stable. The conventional AMCL had a big estimation error in the Y direction leading, to more than a 77 mm RMSE and nearly a 34 mm average value, while the average value of the proposed method was around 27% of the conventional algorithm.

**Figure 9:**
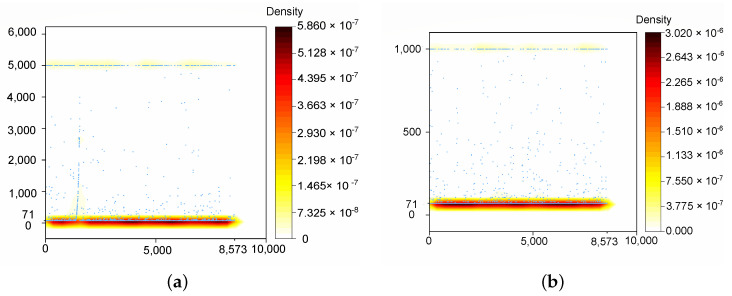
Particle number applied by the conventional AMCL. (**a**) Particle number with respect to sample steps during the whole process with a maximum of 5000 particles. (**b**) Particle number with respect to sample steps during the whole process with a maximum of 1000 particles.

Meanwhile, the particle number of each experiment was monitored for the whole process. The particle number was checked 10 times per second, as shown in Figure 9. For example, the horizontal axis Samplesteps value of 1000 means the 1000th check. For the conventional AMCL and the proposed method, the minimum numbers were both 71, and the maximums were, respectively, 5000 and 1000. The distribution density mainly concentrated at the lowest and highest level. The proportion of the proposed method working with less than 100 particles was 87.8%, while the proportion of the conventional AMCL working with less than 500 particles was 83.7%. When the conventional AMCL was limited to working with 1000 particles, the robot kidnapping problem occurred more frequently. Since each particle represents an iteration, the experiment results imply the efficiency of the proposed method.

### 4.2. Robot Kidnapping Problem

**Figure 10:**
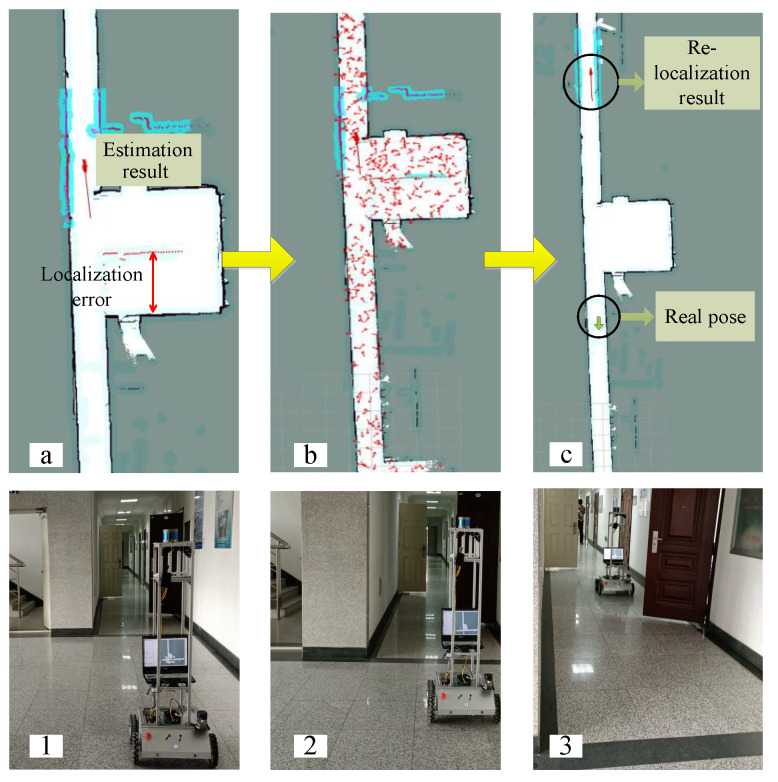
Kidnapping problem with conventional AMCL. (**a**)–(**c**) demonstrate the particle distribution when kidnapping problem happens. (**1**)–(**3**) demonstrate the robot’s real pose respectively.

**Figure 11:**
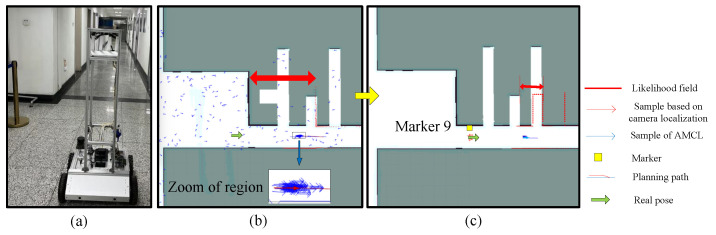
Kidnapping problem with proposed method. (**a**) is the real pose in the environment shown in (**b**). (**c**) illustrates the estimation deviation when the marker is not recognized.

In this section, experiments are executed to examine the system’s capability to solve the robot kidnapping problem. Figure 10 demonstrates the performance of the conventional AMCL, and the proposed method is shown in Figure 11. In Figure 10a, LiDAR measurements find the localization error. In Figure 10b, the threshold of the particle number is improved due to the high-level measurement uncertainty. However, due to the elimination of the right pose in the convergence stage, the system was wrongly localized, as shown in Figure 10c. Due to the repetitive features in the environment, the LiDAR measurements matched well with the local map at the wrong pose; the uncertainty level was considered to be low, and few particles were maintained for position tracking locally. The algorithm was unable to recover from the failure automatically.

Figure 11a is the real state of Figure 11b; the localization error was detected by the likelihood field. Particles were added to correct the errors; blue arrows are the samples of the conventional AMCL. Since there was no visual landmark before the robot arrived at Marker 9, the algorithm kept tracking the clustered blue particles in the zoom. Though part of the localization error was corrected, it was far from a successful recovery. When Marker 9 was recognized, specific particles centered at the visual localization result were generated, as shown in Table 2.

The automatic navigation experiment of both approaches was implemented 10 times in the environment along $P_{1}-P_{33}$, sequentially, and $P_{33}-P_{25}-P_{9}-P_{1}$. The proposed method can recover from the failure automatically when the landmark is perceived. There were six times the conventional AMCL failed to reach $P_{33}$ and was unable to recover. In the other four experiments, the failure happened when returning to $P_{1}$.

## 5. Discussion

In this paper, a low-cost localization method fusing vision data with a modified PF was proposed and verified. The scheme was designed to solve all indoor localization problems with high accuracy and efficiency in long-term performance. The absolute localization results of vision sensors were utilized discontinuously for the correction of the probabilistic estimation. The RMSE and average value of the localization error were used to evaluate the position tracking ability during a 600 m tour. The RMSEs in the X and Y directions were reduced to 76.25 mm and 77.26 mm from 269.92 mm and 285.19 mm, and the average errors in the two direction were reduced by about 73%. Meanwhile, the particle number of the set was monitored, and the experiment showed that the proposed method performed well with 20% of the particles of the conventional AMCL, validating the efficiency of the algorithm. The superiority of the global localization of the proposed method was examined using the robot kidnapping problem.

## Figures and Tables

**Table 1:** Root-Mean-Squared Errors (RMSEs) of the algorithms in position estimations with respect to the ground truth reference.

Item	Conventional AMCL	Proposed Method
RMSE of X (mm)	269.92	76.25
RMSE of Y (mm)	285.19	77.26
Mean of X (mm)	32.03	9.05
Mean of Y (mm)	33.85	9.11

**Table 2:** Performance of solving the kidnapping problem.

Method	Maximum Number	Failure Rate	Recovery Rate
Conventional AMCL	5000	100%	0
Proposed Method	1000	20%	100%

## Data Availability

Not applicable.

## Nomenclature

$u_{t}$	Control data at moment *t*
$C_{t}$	Visual based pose vector at moment *t*
$z_{t}$	Measurement data at moment *t*
$\chi_{c}^{\left[i\right]}$	The *i* th visual based particle
$x_{c}$	Visual based localization result
$\chi_{temp}$	Temporary sample vector
$x_{t}$	Robot’s state at moment *t*
$\chi_{t}$	Current sample vector
$bel(x_{t})$	Posterior belief distribution
*d*	Euclidean distance between $x_{c}$ and $x_{t}$
$\omega_{t}^{\left[i\right]}$	Importance factor of the *i*th $x_{t}$
σ	Threshold for correction of relative localization
$\omega_{fast}$	Short term measurement likelihood
$\alpha_{fast}$	Decay rate of $\omega_{fast}$
$\omega_{slow}$	Long term measurement likelihood
$\alpha_{slow}$	Decay rate of $\omega_{slow}$
$\omega_{avg}$	Average particle weight
ϵ,δ	Statistical error bounds
*m*	Grid map
κ	Parameter in proportion to non-empty state space
$M_{\chi }$	Maximum of particle number according to KLD
$z_{1-\delta }$	Upper (1−δ) quantile of the standard normal distribution

## Abbreviations

The following abbreviations are used in this manuscript:

AMCL	Augmented Monte Carlo
LiDAR	Light Detection and Ranging
PF	Particle Filter
HOG	Histogram of Oriented Gradients
PnP	Perspective-n-Point
KNN	K-Nearest-Neighbor
KLD	Kullback–Leibler Divergence
